# Use of a Flexible Two-Dimensional Textile Dosimeter with a Kilogray Dose Range to Measure the Dose Distribution for a ^60^Co Source

**DOI:** 10.3390/ma18122685

**Published:** 2025-06-06

**Authors:** Marek Kozicki, Radosław Wach, Elżbieta Sąsiadek-Andrzejczak, Piotr Maras

**Affiliations:** 1 Department of Mechanical Engineering, Informatics and Chemistry of Polymer Materials, Faculty of Materials Technologies and Textile Design, Lodz University of Technology, Żeromskiego 116, 90-543 Lodz, Poland; elzbieta.sasiadek@p.lodz.pl; 2 GeVero Co., 90-980 Lodz, Poland; 3 Institute of Applied Radiation Chemistry, Faculty of Chemistry, Lodz University of Technology, Wroblewskiego 15, 93-590 Lodz, Poland; 4 Department of Radiotherapy Planning, Copernicus Hospital, Pabianicka 62, 93-513 Lodz, Poland; piotr.maras@wp.pl

**Keywords:** NBT-cotton dosimeter, textile dosimeter, 2D dosimetry, ionizing radiation, ^60^Co dosimetry

## Abstract

The two-dimensional (2D) measurement of radiation dose distribution on non-planar surfaces requires the use of a flexible dosimeter. This work concerns the use of a unique cotton textile-based dosimeter to characterize the dose distribution of a ^60^Co source used in the research and sterilization of products. Alternatively, for high-dose-rate experiments, an electron beam accelerator has been used. The dosimeter was prepared by the padding-squeezing-drying of a cotton textile made of cellulose, where a 10% solution of nitrotetrazolium blue chloride (NBT) was used for the padding process. NBT served as a radiation-sensitive compound, which transformed into a purple-brown NBT formazan upon exposure to ionizing radiation. The NBT dosimeter is scanned after irradiation using a flatbed scanner, and the data is processed using dedicated software packages, which together constitute a 2D dose distribution measurement system. The green channel of the RGB color model contributes the most to the color change of the dosimeter. The calibration relation obtained for the green channel showed that the dosimeter responds to doses of 0.8–45 kGy. Conversions of the green channel signal were performed using the calibration relation to analyze the 2D dose at a large distance and close to a ^60^Co source shielded by a solid metal and a cylindrical metal structure with holes. Additionally, the dose distribution was assessed using a dosimeter placed on metal implant models undergoing radiation serialization. This work demonstrates the potential of such a dosimeter for characterizing high-dose-rate ^60^Co sources and measuring the dose distribution on non-planar surfaces.

## 1. Introduction

Monitoring ionizing radiation is essential to protect the world and its inhabitants from the effects of improper use of radiation sources or harmful accidents. The impact of healing ionizing radiation effects includes healthcare costs, cleanup efforts at nuclear accident sites, reduction of workforce productivity due to disabilities or deaths, insurance payouts, or compensation for affected individuals, investment in medical countermeasures, protective equipment, and radiation detection technologies [1,2,3]. For example, the accident at the Fukushima Daiichi nuclear power plant causes significant costs related to: (i) support for accident evacuees—$50 billion, (ii) offsite decontamination—~$25–51 billion, (iii) onsite decommissioning—~$20 billion, (iv) replacing power from idled nuclear plants—~$100 billion, and (v) other costs—~$215 billion for image reputation, and $10 billion for loss of tourism [4]. To monitor ionizing radiation, various types of dosimeters have been developed [5,6,7,8,9,10,11,12,13]. In this work, we present 2D dosimeters with a unique structure that is based on natural cellulose fibers and a radiation-sensitive compound—cotton fabric modified with nitrotetrazolium blue chloride (NBT). The dosimeter is flexible, which means it can adapt to the shapes of objects, allowing the measurement of surface dose distribution. In terms of 2D measurement capabilities, this dosimeter is somewhat similar to the very frequently used 2D film dosimeters [11,12], though it is not as flexible as the NBT-cotton dosimeter. In consequence, the use of film dosimeters is mainly limited to flat surfaces.

Two-dimensional film dosimeters are plastic sheets containing radiation-sensitive compounds in their structure that respond to irradiation by changing color. The intensity of the color is related to the absorbed dose, e.g., poly(vinyl alcohol) films reacting in the kilogray dose range with tetrazolium salts as color-forming compounds [13,14,15,16,17,18,19,20,21]. Other similar dosimeters are made of polyvinyl butyral, nitro-blue tetrazolium [22], or methyl red [23]. In turn, for measurements in the low dose range, e.g., radiotherapy dose range, other dosimeters can be used [24,25].

Research on fiber and textile-based dosimeters has been conducted for about fifteen years. In 2011, we first proposed a 2D dosimeter based on polyamide fabric modified with 2,3,5-triphenyltetrazolium chloride (TTC). The absorbed dose was recorded based on the color change of the dosimeter; it turned red when exposed to UV light. The non-uniform exposure of the dosimeter made the changes immediately visible to the naked eye. Several modifications were also made to the dosimeter to obtain a more moisture-resistant system. Other compounds, such as NBT, have also been tested as radiation-sensitive components of polyamide textiles. The TTC-polyamide dosimeter was shown to be superior to NBT-polyamide in terms of UV dose sensitivity; however, its dynamic dose response range is lower than that of NBT-polyamide. Such dosimeters also respond to ionizing radiation, as shown for TTC-polyamide irradiated with ^192^Ir used in cancer brachytherapy. Due to the unique structure of dosimeters, i.e., textile materials with a specific warp and weft, their structure affects the accuracy of dose distribution measurements. Therefore, appropriate flatbed scanning parameters should be used to reduce image noise and improve dose distribution measurements [26]. Apart from modifying textiles to make them sensitive to radiation, it is possible to produce microfibers doped with compounds sensitive to UV and ionizing radiation. Such fibers can be used in the production of textile materials as dosimeters, as presented elsewhere. We also used various textile modification techniques, such as screen printing, to reduce the use of chemicals. Using this technique, it has also been shown that multi-colored patterns can be printed on textile materials, serving both for decorative purposes and as UV sensors and dosimeters.

A flexible, shape-conforming NBT-cotton dosimeter has recently been used for 2D dose distribution measurements, but only for irradiation with artificial UV light generated by UV irradiators [27] and ionizing electron radiation at 6 MeV using an accelerator [28]. The following conclusions were drawn from a previous study [27]: (i) a dynamic dose response of about 2000 mJ/cm^2^, (ii) the green channel of the RGB color space was selected as the one most influencing the color change of the samples, (iii) the intensity of purple-brown color changes (initially a yellowish color) was related to the UVC dose using a second-order exponential decay function, as follows: Green channel = 54.499 ± 2.938 × exp(−Dose/45.769 ± 5.392) + 111.124 ± 3.321 × exp(−Dose/751.807 ± 31.149) + 72.353; however, this relationship can be divided into two dose–response ranges: 5–95 mJ/cm^2^ and 100–2000 mJ/cm^2^; linear and polynomial calibration equations were derived for these dose subranges [27], (iv) the stability of NBT-cotton samples without exposure to sunlight assessed for 10 days showed an approximately 1.4% increase in color with respect to the initial color of the samples, and (v) it was proven that, in the case of non-uniform irradiation of NBT-cotton, it is possible to obtain a 2D dose distribution after scanning the samples with a flatbed scanner and processing the data using the polyGeVero-CT software package (GeVero Co., Lodz, Poland). The study of NBT-cotton dosimeters for ionizing electron radiation revealed the following [28]: (i) dose response of up to ~80 kGy, which can be described by the equation of the Green channel = 59.99 + 95.95 × exp(−Dose/100.87) + 60.25 × exp(−Dose/3.96), (ii) independence of dose response from dose rate for 1.1–73.1 kGy/min, (iii) dose resolution in the range of −0.07 to −0.4 kGy for ~0.6 to ~75.7 kGy for filtered images, (iv) similar to UV-irradiated samples, two dose–response subranges of ~0.6 to ~7.6 kGy and ~9.9 to ~62.0 kGy, and (v) the ability to measure 2D dose distribution. In addition, the NBT-cotton dosimeter has been shown to be useful for measuring 2D dose distribution on rectangular, cylindrical, and sharp triangular surfaces.

**Figure 1:**
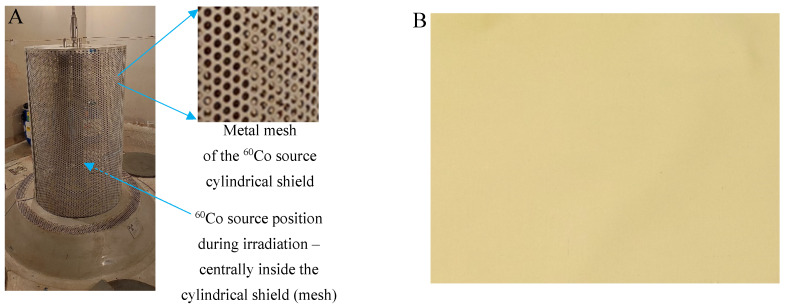
(**A**) ^60^Co source located at the Lodz University of Technology (Lodz, Poland). (**B**) NBT-cotton dosimeter just after preparation.

To date, the NBT-cotton dosimeter has never been evaluated for 2D dose distribution measurements when ^60^Co was used as the ionizing radiation source. This paper describes this type of research. The ^60^Co source is used in the radiation chamber located at the Institute of Applied Radiation Chemistry of the Lodz University of Technology (Poland). The chamber is used for basic and applied research and product sterilization. It is important to know the dose distribution in this chamber under different irradiation conditions, taking into account the location of samples and the surface dose for products with different shapes and structures. The chamber operates by automatically charging the ^60^Co source from the ground to the device located in the chamber (see Figure 1). The device is surrounded by a cylindrical metal mesh shield. Samples to be irradiated can either be attached to the shield or placed at some distance from the shield. Therefore, this study proposes the following scope of research using the NBT-cotton dosimeter and its inherent and unique feature of flexibility and adaptability to various shapes: (i) calibration of the NBT-cotton dosimeter at various positions relative to the ^60^Co source, (ii) measurement of the 2D dose distribution on the surface of the metal shield of the ^60^Co source, and (iii) measurement of the 2D dose distribution on the surface of objects such as medical implants, which are sterilized. The NBT-cotton dosimeter is coupled with 2D scanning using a flatbed scanner and the polyGeVero^®^-CT and polyGeVero^®^ software packages (GeVero Co., Lodz, Poland) for post-scanning data processing. The hypothesis of this work is that NBT-cotton can be used to measure 2D dose distribution on non-flat surfaces when ^60^Co is used for irradiation.

## 2. Materials and Methods

### 2.1. Preparation of Samples

Cotton fabric with the following properties was selected for the preparation of dosimetric samples: white, woven fabric, not brightened, warp: 240/dm, weft: 220/dm, twill weave, surface weight: 250 g/m^2^, and thickness of 0.68 mm (Royal TenCate, Nijverdal, The Netherlands). It was pre-prepared by washing (30 min at 40 °C) in 1 g/dm^3^ of non-ionic surfactant (Rokafenol N8-P7, Boruta Zgierz, Zgierz, Poland) to remove impurities remaining after its production and during storage. After washing, the cotton fabric was thoroughly rinsed with tap and distilled water and, finally, dried at 40 °C for about 120 min and ironed.

The periodic modification method was applied to cotton fabric using a padding machine (E. Benz, Stuttgart, Germany). In this modification method, a 10% nitrotetrazolium blue chloride solution (NBT, M = 817.64 g/mol, Roth, Karlsruhe, Germany) was poured between the rotating and pressed shafts of the machine. Cotton samples were passed through this solution to soak, and then wet samples were squeezed out (clamp 45 kG/cm, rotation speed 4 m/min). Soaking and squeezing were repeated three times. The next process was drying the NBT-modified cotton samples at 30 °C for 120 min. The samples were then covered with aluminum foil to protect from daylight and enclosed in polyethylene–polyamide sleeves to protect against moisture and stored in a refrigerator at a temperature of approximately 4 °C until irradiation. NBT-cotton samples stored in this way retained their natural yellow color (Figure 1B) for at least a month. The accuracy of NBT solution preparation was reduced to the accuracy of a 100 mL volumetric flask (±0.1 mL) and laboratory scale: ±0.1 mg (model: AS220.X2 PLUS, RADWAG, Radom, Poland). Each stage of preparation of the NBT cotton dosimeter, before chemical modification, after soaking, squeezing, and drying, resulted in a change in its weight: 2.3250, 5.4831, 3.4421, and 2.4832 g, respectively (for a sample with an area of 10.0 ± 0.1 cm^2^). NBT cotton samples were prepared in several dimensions due to further planned experiments in this study: (i) approximately 2 × 3 cm^2^, which served as calibration samples, and (ii) other, maximally ranging from approximately 10 × 10 cm^2^ to 15 × 15 cm^2^, which were used to examine the dose distribution in various places in relation to ^60^Co and on the surface of sterilized implants.

### 2.2. Stability of Samples

NBT-cotton samples were prepared as described in Section 2.1; however, a 1% NBT solution was used to produce them. Then, the samples were stored in various conditions for 150 days: (i) at a temperature of approximately 20–23 °C with access to daylight, (ii) at a temperature of approximately 20–23 °C covered with aluminum foil, (iii) at about 2–4 °C covered with aluminum foil, and (iv) at about −17–−20 °C covered with aluminum foil. Samples were measured periodically using a reflectance spectrophotometer (Spectraflash 300, D65⁄10; 10 nm resolution; measurement error of 0.1%; DataColor, Rotkreuz, Switzerland). The device was calibrated beforehand, and the UV light of 190–400 nm was automatically cut off by the software (microMATCH v. 3.6; DataColor) after the 0% UV option was selected to avoid unnecessary irradiation of the samples. The reflectance at 550 nm was analyzed for stored samples compared to samples just after preparation.

### 2.3. Irradiation of Samples

Samples were irradiated with the ^60^Co radioisotope used in a radiation chamber installed at the Institute of Applied Radiation Chemistry, Lodz University of Technology, Poland (Ob-Servo-D dry storage multipurpose panoramic Co-60 irradiator, CoS-44 HH-N type, installed in 2014, nominal activity of 2200 TBq (60 kCi)) produced by the Institute of Isotopes Co., Ltd., Budapest, Hungary). This facility is used for both research related to radiation chemistry and the sterilization of products. NBT-cotton dosimeter samples were fixed in different positions relative to the source and the metal mesh shield of the source (Figure 1): (i) attached to the metal mesh shield in such a way that the sample (approximately 2 × 3 cm^2^) is located mainly between the holes of the shield, for calibration purposes, (ii) attached to the metal mesh shield in such a way that the sample (approximately 2 × 3 cm^2^) mainly covers the holes of the shield, for calibration purposes, (iii) attached to the shield for 2D dose distribution analysis (larger samples from approximately 10 × 10 cm^2^ to 15 × 15 cm^2^), and (iv) attached to metal implant models in such a way that the samples cover their surface, for dose distribution analysis on their surfaces in different locations.

The absorbed dose was also measured on the implants—metal bars with a diameter of 16 and 8 mm (on the front and back of the bars), exposed to the ^60^Co radiation source as described above. The bars were wrapped with an NBT-cotton dosimeter enclosed in polyamide-polyethylene sleeves (vacuum-packed), placing the dosimeters at the height of the center of the ^60^Co source. The following implants/bars, were used: (i) Ti & TiNb, diameter: 16 mm, 11 cylinders, 3 mm-high (packed to form a solid cylinder), (ii) 316L steel, diameter: 16 mm (dose measured with a film dosimeter, see below: 33.6 kGy front side, 12.4 kGy rear side), (iii) CoCrMo, diameter: 16 mm (dose measured with a film dosimeter: 34.8 kGy front side, 11.5 kGy rear side), (iv) TiTa6V, diameter: 8 mm (dose measured with a film dosimeter: 18.0 kGy front side, 18.0 kGy rear side), (v) ASTM F136—alpha-beta titanium alloy with aluminum and vanadium, (vi) TiAl6Nb7, diameter: 8 mm, (vii) ASTM F1537, alpha-beta titanium alloy with aluminum and vanadium, and (viii) CoCrMo, diameter: 8 mm.

One sample was placed in a wedge (P4701 Risø Aluminum Energy Wedge, GEX Corporation, Palm City, FL, USA) and irradiated with ^60^Co. The wedge is most commonly used for the IQ/OQ testing of electron dose distribution and energy measurement at electron beam (EB) facilities. It is made of aluminum and has dimensions of 12 cm (L), 14 cm (W), and 2.9 cm (H) (±0.1 mm). It consists of two triangular parts (16.0 ± 0.3°) that, together, form a cuboidal-shaped wedge. A film dosimeter or NBT-cotton dosimeter is placed between these two parts prior to irradiation.

A linear accelerator set to operate in 4 µs pulses at 20 Hz to generate 6 MeV electrons (ELU 6-E Elektronika, Moscow, Russia) was also used for irradiation of NBT-cotton samples. The samples were irradiated to the same dose of 30.8 kGy at different conditions: (i) vacuum-packed and kept in packaging also after irradiation, (ii) vacuum-packed and opened immediately after irradiation to expose them to air, and (iii) irradiated in air (all samples were scanned 6 h after irradiation, Section 2.4). The samples were positioned perpendicular to the electron beam at 160 cm distance from the beam outlet, and the irradiation took 6 min.

The measurements of radiation dose with the NBT-cotton dosimeter were associated with 1D measurements of absorbed dose using film dosimeters (B3 WINdose Dosimeters, average thickness: 0.0190 ± 0.0003 mm, GEX Corporation, Palm City, FL, USA).

### 2.4. 2D Scanning of Samples

All dosimeter samples were scanned using a flatbed scanner (Epson L395/L3050, Suwa, Japan). The settings were: 75 dpi color images (BMP), unsharp mask (off), color restoration (off), shadow correction (off), moiré removal (off), dust removal (off), brightness (0), contrast (0), and saturation (0).

### 2.5. Data Processing

Bitmaps of NBT-cotton samples after scanning with a flatbed scanner were processed using the polyGeVero^®^-CT (v. 1.2, GeVero Co., Lodz, Poland) software package. The images were analyzed in the RGB color model to decide which channel (red, green, or blue) contributed the most to the color changes of the samples after irradiation. It was concluded that all channels contribute significantly to color changes, with the green channel having the largest contribution; therefore, it was selected for further calculations. To smooth the images and reduce the impact of the fabric structure on the data analysis, filtering of images was applied: mean filter, kernel size: 3, kernel unit: mm, kernel mode: 2D, iterations: 2. The images were processed into 2D dose distribution maps using the calibration equation, and exported to the polyGeVero^®^ software package (v. 2.0, GeVero Co., Lodz, Poland) for further analysis [29].

## 3. Results and Discussion

### 3.1. Storage and Calibration of NBT-Cotton

The stability of NBT-cotton samples after preparation depends on storage conditions (Table 1). Exposure to daylight at room temperature caused the greatest changes in the color of NBT-cotton, which, after 150 days of storage, increased by over fifty percent compared to the initial sample. The difference between samples 1 and 2 (Table 1) is the access to daylight; sample no. 2 was protected from daylight. This resulted in a significant reduction in color change, indicating that daylight plays an important role in the instability of the samples. The second factor is storage temperature (samples 3 and 4). Lowering the storage temperature significantly improves the long-term stability of samples. It is enough to store samples in the refrigerator to achieve more than eight times smaller color changes over 150 days of storage. Interestingly, lowering the temperature to approximately −20 °C (in the freezer) did not improve stability compared to samples stored at 2–4 °C for the first 30 days. Only after longer storage were the samples more stable compared to storage in the refrigerator. The reason for the slightly lower stability of samples stored in the freezer is unknown, but it may be related to the rapid crystallization of water from the moisture contained in the sample volume. In summary, all NBT-cotton samples were stored in a refrigerator at about 2–4 °C after both manufacturing and irradiation. A detailed study on the stability of the NBT-cotton dosimeter was recently published [30].

**Figure 2:**
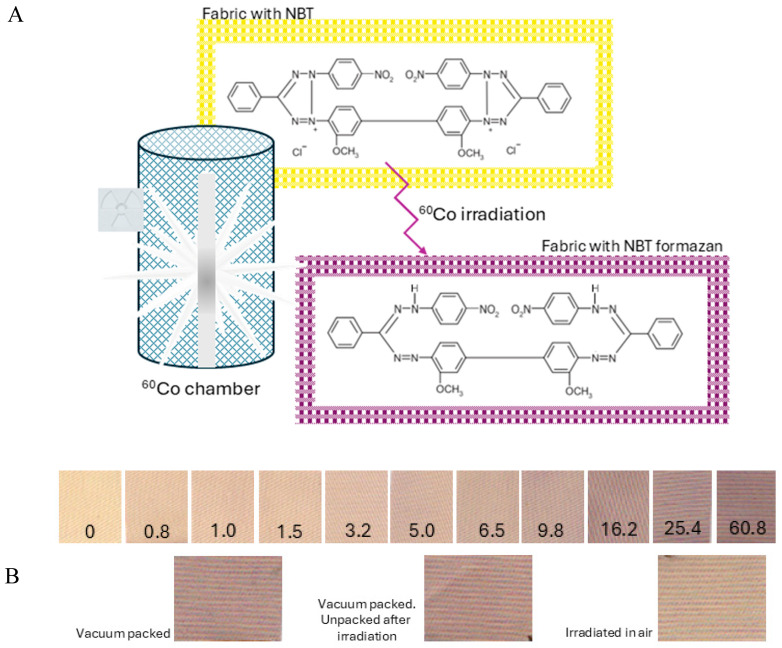
Scheme of the reaction occurring during irradiation of the NBT-cotton samples with ^60^Co (**A**) and samples after irradiation (**B**), vacuum-packed in polyethylene–polyamide sleeves (first line), and a comparison of samples irradiated to the same dose of 37.2 kGy with 6 MeV electrons (second line, scanned 6 h after irradiation, irradiation time = 6 min): vacuum-packed and kept in packaging, vacuum-packed and unpacked 1.5 min after irradiation, and irradiated in air (from left to right); doses (kGy) are given in the photos.

Irradiation of NBT-cotton with ^60^Co or an electron beam causes a color change from yellowish to purple-brown, with the intensity depending on the radiation dose (Figure 2). The reason for the color change is the formation of NBT formazan in the structure of the cotton fabric (Figure 2). The conditions under which samples are irradiated and stored after irradiation affect the color of the sample (Figure 2B). A very distinct difference in color was observed between samples irradiated in air and those protected from air by vacuum-packing in polyamide-polyethylene sleeves. Vacuum-packed samples were characterized by a greater intensity of purple-brown color than samples irradiated in air (Figure 2B). The mean values of the green channel of the RGB color model for the sample vacuum-packed and irradiated, vacuum-packed and opened just after irradiation, and irradiated in air are 130.59 ± 11.17, 133.57 ± 12.70, and 155.27 ± 11.83 [-], respectively. These observations lead to the conclusion that oxygen from the air affects the color of the sample, causing a color of lower intensity compared to the vacuum-packed and irradiated sample, due to competition between the oxidation of the NBT substrate (lower intensity of color or bleaching of color) and its reduction [14] leading to the formation of purple-brown formazan. It seems that opening the package with NBT-cotton right after exposure has no clear effect on the color intensity of NBT-cotton.

**Figure 3:**
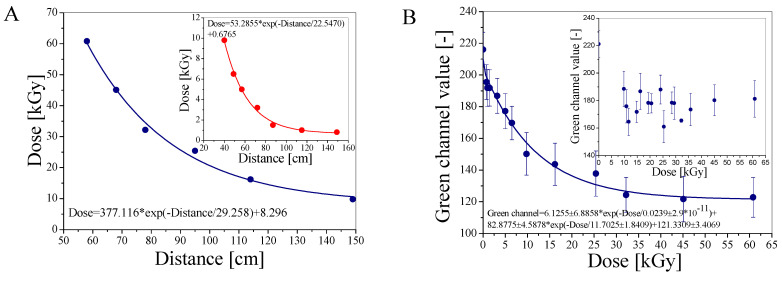
Dosimetry of ^60^Co source using B3 WINdose dosimeters (GEX Corporation, USA) at different distances from the source: 58–149 cm at irradiation time 65 h (**A**) and 40–149 cm at irradiation time 4 h (inset in (**A**)). Calibration of NBT-cotton for the samples irradiated at the same distances from ^60^Co as in (**A**). Samples were vacuum-packed in polyamide–polyethylene sleeves (inset: samples exposed to air during irradiation) (**B**). Two batches of NBT-cotton samples were irradiated at distances 40–149 cm for the dose range of 0–9.8 kGy and 9.8–60.8 kGy at irradiation time 4 and 65 h, respectively (sample kept in a fridge after irradiation; sample removed from sleeves 8 h after irradiation and scanned; sample irradiated with a dose of 9.8 kGy for 4 h).

NBT-cotton samples were scanned after irradiation with a flatbed scanner, and the obtained images (BMP files) were resolved to analyse the influence of the red, green, and blue channels of the RGB color model on the color formation. The green channel contributed the most to the color changes, so its intensity was then analysed in relation to the radiation doses. In parallel, B3 WINdose film dosimeters (Figure 3A) were irradiated together with NBT-cotton at the same distances from the ^60^Co source. Finally, a calibration graph for NBT-cotton (green channel value versus radiation dose, Figure 3B) was prepared for the doses from the film dosimeters. The calibration relationship for NBT-cotton is given in Figure 3B. The NBT-cotton dosimeter responds to doses of approximately 0.8–60.8 kGy. Its saturation begins at approximately 32 kGy. It is also obvious that the dosimeter samples must be protected from air (oxygen) to obtain an adequate response to irradiation; exposure to air during irradiation results in a poor dose response, as seen in the inset of Figure 3B. Therefore, all other NBT-cotton samples were vacuum-packed in polyamide-polyethylene sleeves for irradiation and then unpacked for 2D measurements using a flatbed scanner. It can also be determined from the calibration graph that the dose measurement using NBT-cotton is associated with a significant error (standard deviation, Std). This is related to the structure of the dosimeter, which is a cotton textile with a twill weave, which affects the scan quality and the Std values for the calibration points. Consequently, this is propagated in the 2D dose distribution measurements (reported in the next sections). One way to alleviate this adverse effect in the future is to produce dosimeters with other weaves to obtain smoother surfaces. Applications of the dosimeter for 2D dose measurements included irradiating dosimeter samples under various conditions, scanning, and processing the data, which constituted the conversion of green channel values to dose using the second-order exponential decay calibration shown in Figure 3B, as follows: Green channel = 6.1255 ± 6.8858 × exp(−Dose/0.0239 ± 2.9 × 10^−11^) + 82.8775 ± 4.5878 × exp(−Dose/11.7025 ± 1.8409) + 121.3309 ± 3.4069. A linear dose range is estimated up to approximately 10 kGy; a linear regression is as follows: Green channel = −5.51 × Dose + 204.03 (R^2^ = 0.92), indicating a dose sensitivity of −5.51 1/Gy. The results of the application study are presented in the following sections.

### 3.2. Two-Dimensional Dose Distribution Measurements near ^60^Co Shield

**Figure 4:**
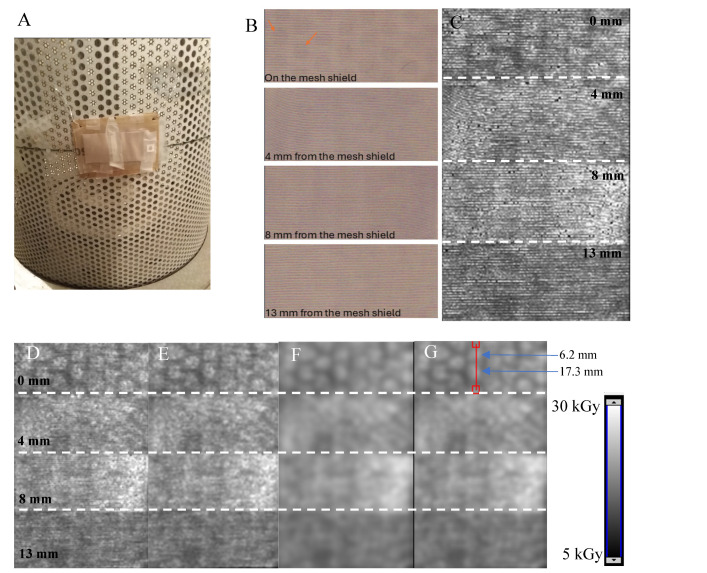

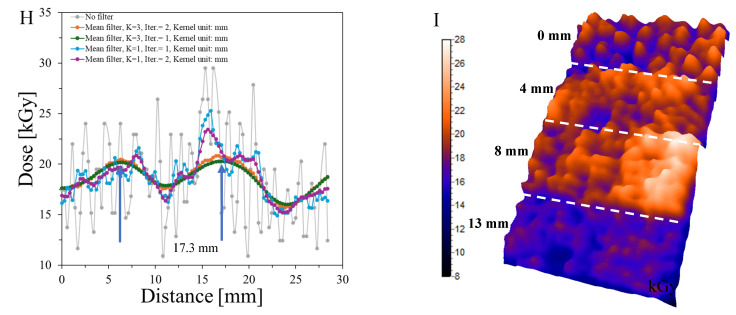
Irradiation setup and results for NBT-cotton wrapped in polyethylene–polyamide sleeves and exposed to ^60^Co. Figure (**A**) is a photograph of the samples attached to the mesh shield of the ^60^Co source at the position corresponding to the middle of the source. The package of the sample is made such that the distance of the samples from the shield was about 0, 4, 8, and 13 mm. In (**B**), the samples after irradiation are presented. The orange arrows point to the darker circular regions where the samples were irradiated to higher doses due to holes in the mesh shield. (**C**–**G**) represents the samples as in (**B**), but after scanning with a flatbed scanner, recalculations to dose distribution using the calibration equation shown in Figure 2B (**C**), and after applying the mean filter with the following settings: Kernel = 1, Kernel unit: mm, no. of iterations = 1 (**D**), Kernel = 1, Kernel unit: mm, no. of iterations = 2 (**E**), Kernel = 3, Kernel unit: mm, no. of iterations = 1 (**F**), and Kernel = 3, Kernel unit: mm, no. of iterations = 2 (**G**). In (**H**), dose profiles are shown for all the samples, both before and after mean filter application. The position of the profiles is indicated by the red line in (**G**) (the length of the red line is 28.41 mm). In (**I**), 2D dose distribution maps are presented for the NBT-cotton samples at different positions from the mesh shield (on the basis of filtered images: Kernel = 3, Kernel unit: mm, no. of iterations = 2). The greyscale in (**G**) is for the images in (**C**–**G**). The blue arrows in (**G**,**H**) indicate the middle of the high-dose circular areas. The white dashed lines are separators of the samples. The irradiation time of the samples was about 8 h.

The design of the ^60^Co chamber allows for irradiation of samples by attaching them to the metal mesh shield of the device (Figure 4A) or by placing them at different distances from the source. The NBT-cotton dosimeter was used to evaluate the effect of the mesh shield on the uniformity of irradiation of all samples irradiated during different experiments or sterilization of objects. It should be emphasized that the dose rate on the shield is the highest in the ^60^Co chamber room. The results presented in Figure 4 correspond to: (i) the NBT-cotton dosimeter samples attached to the shield (Figure 4A)—the sample package is visible, and the distance of the samples from the shield is approximately 0, 4, 8, and 13 mm, (ii) the samples seen after scanning with a flatbed scanner (Figure 4B), (iii) 2D dose distributions (C) after recalculating the images in Figure 4B using the calibration equation shown in Figure 3B, (iv) 2D dose distributions after filtering using a mean filter of different parameters (D–G)—see figure caption, (v) an example dose profile (H) at the position indicated in G, passing through the parts of the dosimeter covering the two holes in the mesh shield located at 6.2 and 17.3 mm from the top of the sample, and (vi) 2D dose distribution maps presented in the 3D view option of the software used for processing the results. The results indicate that the dose distribution measurement method using a chemically modified textile material with scanning by a flatbed scanner and proper data processing is a promising dosimetric tool, despite the obvious disadvantage of the weave structure affecting the quality of the measurements. The mesh shield of the ^60^Co source causes a circular spot pattern on the irradiated NBT-cotton. The example profile indicates a sinusoidal pattern of dose distribution with a peak-to-valley difference of about 2.5 kGy for the samples at 0 mm from the shield. This pattern is visible for NBT-cotton samples at 0–8 mm from the mesh shield. At a greater distance of 13 mm, the dose distribution recorded by the NBT-cotton becomes uniform. This is an important observation to consider when irradiating any samples attached to the shielding. The mean dose measured by NBT-cotton against the B3 WINdose film dosimeter for dosimeters attached directly to the mesh shield in the area of the shield opening is 19.8 ± 0.8 kGy and 24.6 kGy for NBT-cotton and the film dosimeter, respectively. For the shield metal, it is 14.6 ± 0.3 kGy and 19 kGy, respectively.

**Figure 5:**
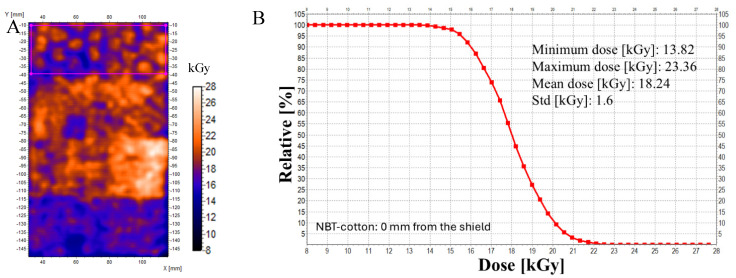

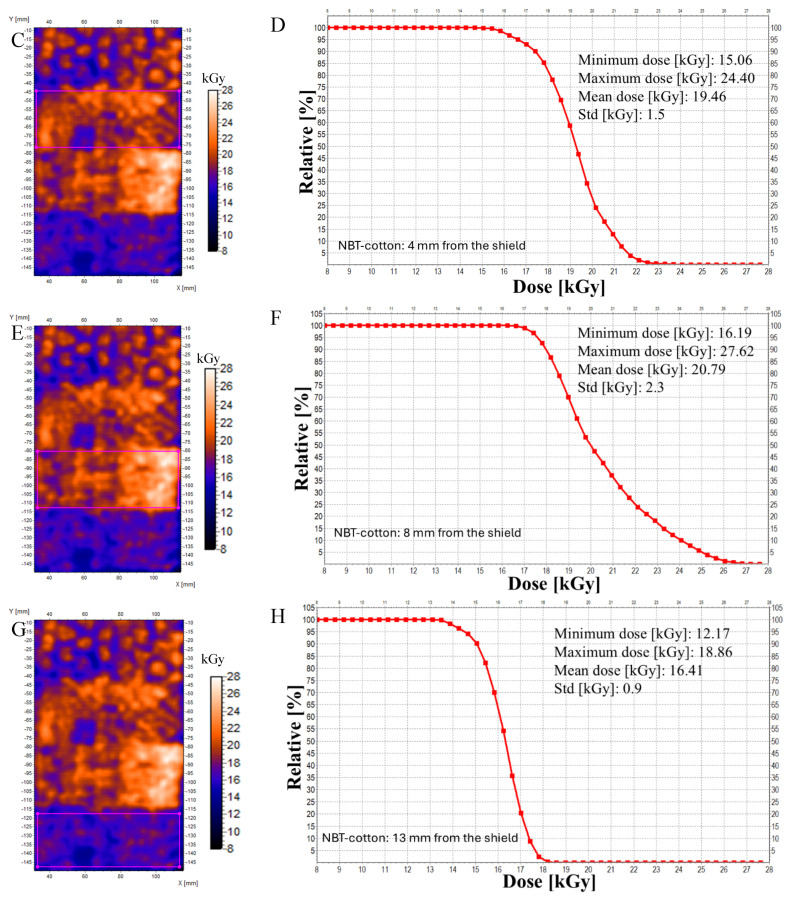
Histograms of 2D dose distribution for NBT-cotton dosimeters (**B**,**D**,**F**,**H**) irradiated with ^60^Co, attached to the mesh shield of the radiation source, as presented in Figure 4, at different distances from the shield: approximately 0, 4, 8, and 13 mm. The histograms have been calculated for the regions of interest (ROIs) indicated in the corresponding 2D dose maps as magenta rectangles (**A**,**C**,**E**,**G**). The results corresponding to each other are (**A**,**B**), (**C**,**D**), (**E**,**F**), and (**G**,**H**). The insets in the histograms are minimum, maximum, and mean doses (mean doses with standard deviations, Std) for the ROIs. Pixel spacing (X and Y) for (**A**,**C**,**E**,**G**) is 0.3 mm. Dose distributions calculated on the basis of filtered images: Kernel = 3, Kernel unit: mm, and no. of iterations = 2.

The minimum, maximum, and mean doses were calculated for almost-whole samples (ROIs are presented in Figure 5A,C,E,G) irradiated at different distances from the ^60^Co source, and dose distribution histograms were calculated (Figure 5B,D,F,H). The corresponding results (mean doses) for the B3 WINdose film dosimeter, however, calculated only on the basis of the circular surface of the dosimeters with a diameter of about 0.5 cm, are 21.8 (maximum dose = 24.6 kGy and minimum dose = 19.0 kGy), 22.8, 20.8, and 18.8 kGy, respectively, for distances of 0, 4, 8, and 13 mm. With some caution, it can be concluded that the results show some similarities; for example, at distances of 8 and 13 mm from the source, the doses decrease, which is recorded by both dosimeters. However, due to the completely different nature of the dosimeters (1D film dosimeter and 2D NBT-cotton dosimeter) and the contribution of inaccuracies due to the textile weave, direct comparison is difficult. The 2D NBT cotton dosimeter provides more information on the non-uniformity in the dose distribution, which cannot be seen with the film dosimeter.

### 3.3. Two-Dimensional Dose Distribution Measurements on Implant Materials

**Figure 6:**
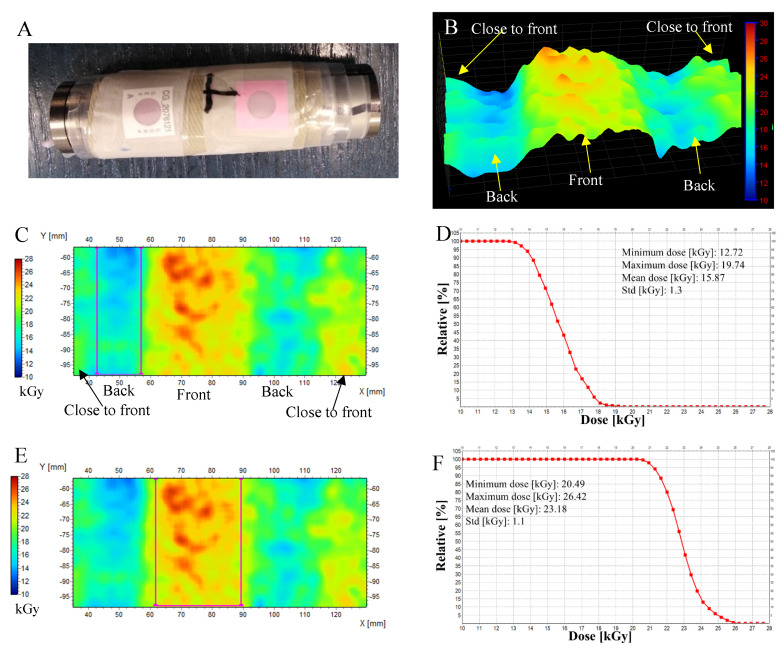

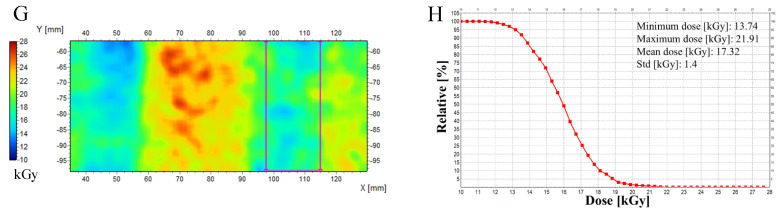
Two-dimensional dose distribution analysis on the TiVa and TiNb metal alloys. (**A**) Eleven cylinders of 3 mm thickness and 16 mm diameter combined together (dose measured with a film dosimeter: 35.2 kGy (front side) and 18.6–18.8 kGy (back side)). (**B**) Three-dimensional view of the 2D dose distribution. (**C**,**D**), (**E**,**F**), and (**G**,**H**) are 2D dose distribution maps and dose histograms corresponding to each other in regions of interest (ROIs), as indicated by magenta rectangles in (**C**,**E**,**G**). Dose distributions calculated on the basis of filtered images: Kernel = 3, Kernel unit: mm, and no. of iterations = 2. Irradiation conditions: dose rate of 730 Gy/h.

**Figure 7:**
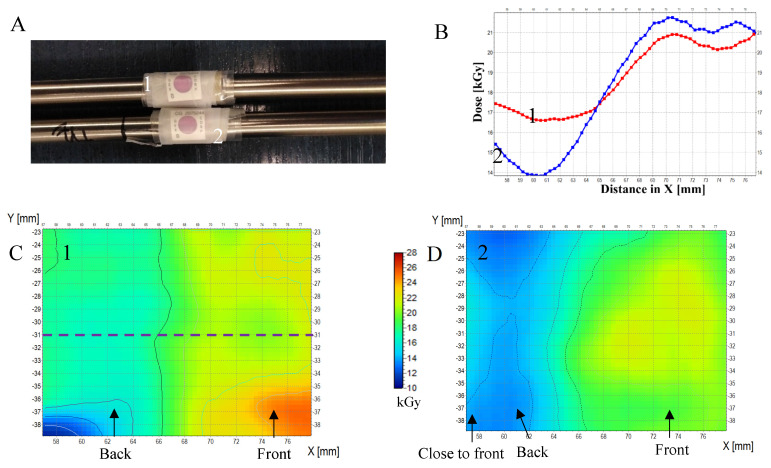
Two-dimensional dose distribution analysis on the following alloys: TiAl6Nb7 (8 mm diameter, ASTM F1537) (**A**)—alloy no. 1 and TiTa6V (8 mm diameter; ASTM F136) (**A**)—alloy no. 2. In (**A**), both alloys are presented with NBT-cotton dosimeter fixed around the alloy samples and film dosimeters attached. In (**B**), profiles along the X-axis, as indicated in (**C**), are presented for NBT-cotton dosimeters after scanning with a flatbed scanner and image conversion to 2D dose distribution maps. The maps are presented in (**C**,**D**) for alloys no. 1 and 2, respectively. The dose measured with the film dosimetry was 34.8 kGy (front side) and 24.9–25.0 kGy (back side). Dose distributions for NBT-cotton calculated on the basis of filtered images: Kernel = 3, Kernel unit: mm, no. of iterations = 2. Irradiation conditions: dose rate of 730 Gy/h.

**Figure 8:**
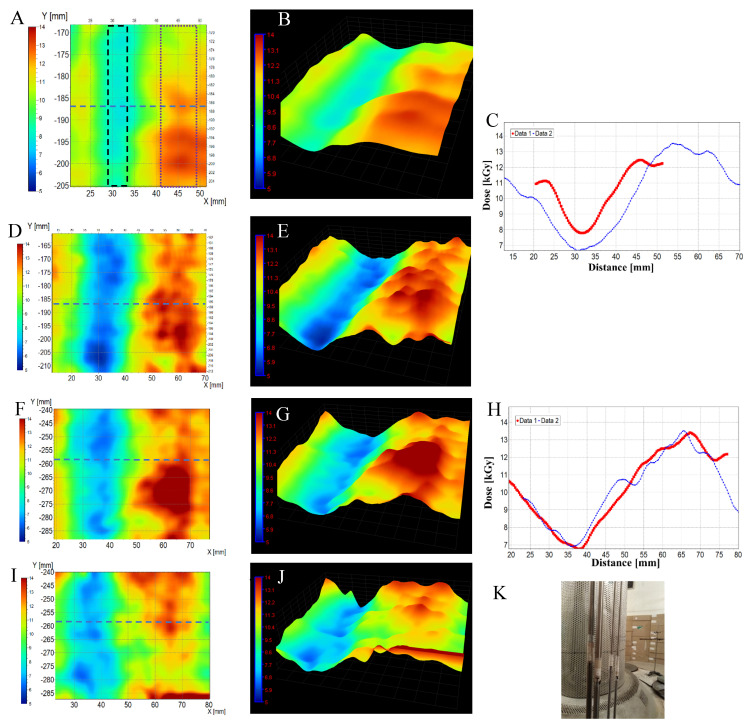
Two-dimensional dose distribution analysis for the following alloys: (**A**,**B**) are for an 8 mm diameter rod made of a CoCrMo alloy, (**D**,**E**) are for the same alloy but with a 16 mm diameter rod, (**F**,**G**) are for a 16 mm diameter rod made of stainless steel (316L), and (**I**,**J**) are for a set of eleven 16 mm diameter and 3 mm-high cylinders made of a TiVa and TiNb alloy. (**C**) represents two profiles along the X-axis at the position shown by the dashed line in (**A**,**D**), corresponding to Data 1 (**A**) and Data 2 (**D**). In (**H**), two profiles are shown along the X-axis at the position indicated by the dashed line in (**F**,**I**), corresponding to Data 1 (**F**) and Data 2 (**I**). The presented 2D dose distributions are based on the filtered green channel of the RGB color model images: mean filter, kernel = 3, kernel unit: mm, no. of iterations: 2. Images in (**B**,**E**,**G**,**J**) correspond to images in (**A**,**D**,**F**,**I**), respectively, and were prepared using the 3D view option of the polyGeVero software package (GeVero Co.) to better visualize the recorded dose distributions. (**K**) shows a photograph of the NBT-cotton dosimeters; the dosimeter samples were attached to rods placed in the ^60^Co chamber. ROIs for back and front sides from which mean dose values were calculated are presented by the black dashed and purple dotted lines in (**A**), respectively. Irradiation conditions: dose rate of 730 Gy/h.

Implantable materials require sterilization. One of the sterilization methods chosen is ionizing radiation due to its inherent penetrating properties. In this respect, gamma radiation produced by the radioactive decay of ^60^Co fulfils this purpose even in the case of metal implant models. Some implants have a specific irregular shape. From the application point of view, the implant should be sterile in every part after irradiation, which means that the dose distribution has to be measured in different parts of the implant. Flexible dosimeters are therefore required. In this respect, it is worth examining textile dosimeters. Therefore, in this section, we present for the first time the application of NBT-cotton for dose distribution measurements for different implants made of metal alloys. The experiment was set in two parts: (i) initially, the possibility of measuring the dose distribution on the surface of TiVa and TiNb (Figure 6), TiTa6V and TiAl6Nb7 (Figure 7) metal alloys was assessed; they were exposed to maximum dose of about 35 kGy, and then (ii) on the surfaces of CoCrMo, stainless steel (316L), and TiVa and TiNb alloys (Figure 8, Table 2), which were exposed to doses even three times lower.

In Figure 6, the results are shown for TiVa and TiNb metal alloys—11 cylinders of 3 mm height (packed in a cylinder) and wrapped with an NBT-cotton dosimeter. Additionally, B3 WINdose film dosimeters were attached to the implants; the dose measured by a film dosimeter: 35.2 kGy (front side) and 18.6–18.8 kGy (back side). Similarly, the dose distribution was measured on the surface of TiTa6V alloy implants, and the results are presented in Figure 7. The dose measured with the film dosimetry was 34.8 kGy (front side) and 24.9–25.0 kGy (back side). The following conclusions were drawn from the first part of the experiment: (i) NBT-cotton allowed the recording of the dose distribution at the front, side, and back of the implants, (ii) the range of measured doses for the front or back side of the implants (regions of interest are shown in Figure 6C,E,G) is wide, which is due to the inhomogeneous structure of the textile weave; this requires further refinement, (iii) NBT-cotton dosimeter changes its color only slightly after irradiation to about 25 kGy and saturates at more than 30 kGy for the irradiation conditions reported in this work (Figure 3); this translates into a lack of agreement with the doses measured by the film dosimeter for the TiVa and TiNb metal alloys at the front of the implant and for the TiTa6V alloy on both sides—the alloys absorbed doses exceeding the measurable dose range of NBT-cotton; the dose distribution at the back of the TiVa and TiNb alloy for NBT-cotton is closer to the point dose measured by the film dosimeter. Decreasing the maximal dose was favorable for the NBT-cotton samples since the maximal measured doses were well below the saturation dose for this dosimeter (Figure 8, Table 2). As in the first part of the experiment, NBT-cotton proved useful for 2D dose distribution measurements on non-planar surfaces, clearly showing decreases in absorbed doses on the backsides of implants compared to the doses on the front sides of implants. The mean doses measured using NBT-cotton were similar to those recorded by the film dosimeter (Table 2). The observed dose differences indicate higher doses recorded by NBT-cotton, which may be due to the long irradiation time at room temperature and the spontaneous color development and non-uniform weave structure of cotton. Overall, although the NBT-cotton dosimeter shows potential for recording dose distribution on non-planar implant surfaces, its structure requires improvements towards the smoothness of ordinary inkjet paper to improve dose distribution recording.

### 3.4. Two-Dimensional Dose Distribution in the Metal Wedge

**Figure 9:**
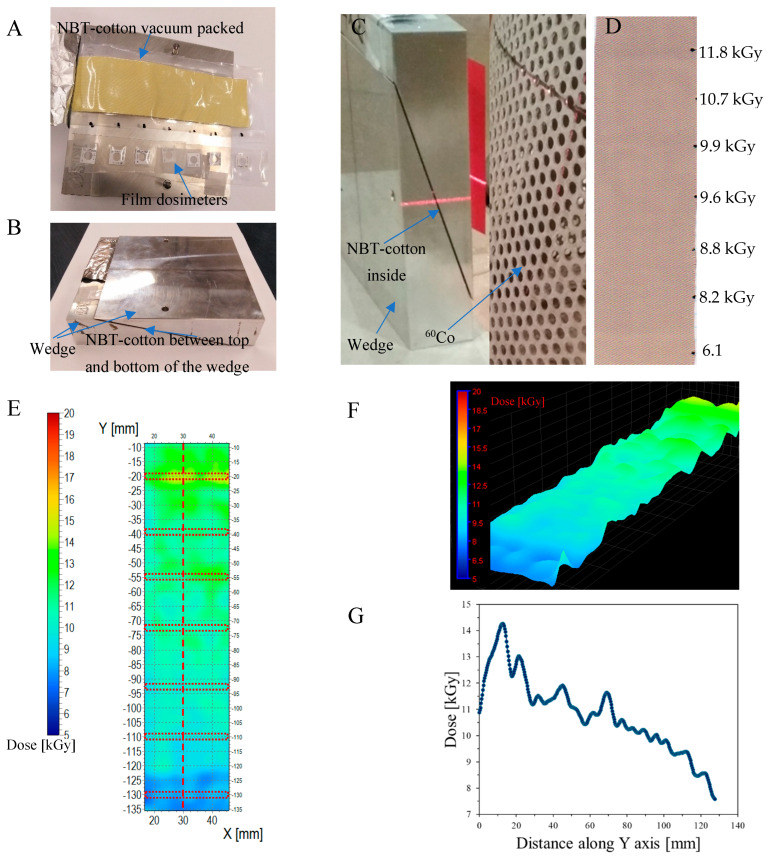
Application of NBT-cotton for 2D dose distribution assessment in a metal wedge. (**A**) NBT-cotton sample placed on the wedge. The sample is in a vacuum-packed polyethylene–polyamide sleeve. Next to it, there are film dosimeters at positions indicated in this figure and in (**D**), with black dots. In (**B**), the NBT-cotton sample is enclosed with the top part of the wedge. Only a part of the sample sticks out of the wedge. In (**C**), the wedge with NBT-cotton is shown during irradiation with ^60^Co. In (**D**), the NBT-cotton sample is presented after irradiation. The doses next to it correspond to the doses read by the film dosimeters. In (**E**), a dose distribution is presented as measured with the NBT-cotton sample. Regions of interest (ROIs) from which the mean dose value was calculated are presented by the red dotted lines at the Y positions −130, −110, −92.5, −73, −55, and −20 mm. In (**F**), the same results are shown as in (**E**), though using a 3D view option for planes using the polyGeVero software package (GeVero Co., Poland). In (**G**), a profile along the Y-axis of the NBT-cotton samples is shown. The location of the profiles is indicated by the red dashed line in (**E**). Dose distributions for NBT-cotton calculated on the basis of filtered images: Kernel = 3, Kernel unit: mm, no. of iterations = 2. Irradiation time was 12.5 h.

A metal wedge, which is used for characterizing ionizing radiation, was used to test the NBT-cotton dosimeter. This wedge consists of two parts that, when placed one on top of the other, form a rectangular metal block (Figure 9A–C). The NBT-cotton sample was placed inside the wedge, as illustrated in Figure 9A,B, and then irradiated with ⁶⁰Co while enclosed in the wedge, as shown in Figure 9C. In this way, the sample dosimeter was exposed to a gradient dose distribution due to the changing thickness of the part of the wedge covering the dosimeter. The intention was to study the response of the sample to irradiation under such conditions. The sample after irradiation is shown in Figure 9D. The black dots on this sample indicate where the B3 WINdose film dosimeters were placed; the doses were read by the film dosimeters (1D measurements). The NBT-cotton after irradiation, scanning with a flatbed scanner, and conversion of the RGB green channel values into dose distribution is shown in Figure 9E,F, whereas in Figure 9G, a dose profile along the sample is presented. In general, NBT-cotton responded to gradient irradiation, and the measurement indicates a decrease in absorbed dose with increasing wedge thickness. However, both the type of cotton weave and the packing of the sample in the polyamide-polyethylene sleeve were responsible for the nonuniformity in the recorded dose distribution. Vacuum-packing of the sample in the sleeve causes a wavy pattern, as seen in Figure 9A. It was found that the method of protecting the NBT-cotton dosimeter material from air (oxygen) should be improved to enhance the smoothness of the samples and eliminate the inaccuracies in the recorded dose distribution (particularly visible in Figure 9G). The doses measured by both dosimeters are compared in Table 3. Both dosimeters showed a similar trend in the measured doses. However, the film dosimeter measures doses only in 1D, and no standard values are given for such a measurement. This is in contrast to the NBT-cotton dosimeter, which is a two-dimensional dosimetry system, and average doses with a standard deviation value can be obtained for any area of interest, providing more information on the dose distribution.

### 3.5. On the Use of NBT-Cotton for ^60^Co and EB Dosimetry—Discussion

The NBT-cotton dosimeter with flatbed scanning and data processing using dedicated software packages constitutes a 2D dosimetry system. So far, the system has been tested for ionizing radiation dosimetry, for EB [28] and ^60^Co irradiation in the current work. There are some similarities and differences between the response of the dosimeter to different types of radiation and radiation conditions. For example, in both types of radiation, the samples transform into the same colors, and the color intensity increases with the increase of the radiation dose. The current work showed that the samples should be protected from air (oxygen) during ^60^Co irradiation, because NBT oxidation occurred in the air, and the color change was not satisfactory. The color saturation for vacuum-packed samples was determined to be over 32 kGy, while for EB irradiation, it was not observed for doses as high as 60 kGy. Furthermore, in the case of EB irradiation, the samples did not need to be protected from air (oxygen), as no concerning problems related to air irradiation were observed. One of the reasons for these observations was the significant differences in the dose rates used for both sources. In the case of gamma (^60^Co) irradiation, the dose rates were in the range of ~0.5–3 kGy/h, and the irradiation times were longer than in the case of EB irradiation with much higher maximum dose rates; dose rate range: 1.1–73 kGy/min. Moreover, the non-uniform EB irradiation of NBT-cotton at a high dose rate resulted in very distinct dose distribution patterns with steep dose gradients (see Figures 5 and 6 in [28]). In the case of such distinct and intensive color changes, the influence of the textile structure and data processing on the image quality was marginal. On the other hand, in the case of non-uniform ^60^Co irradiation (through the source shielding mesh), the dose gradient changes were barely visible, and the cotton fabric weave pattern had a greater influence on the quality of the dose distribution results. To sum up, the NBT-cotton dosimeter in its current form is acceptable for recording EB dose distributions at high dose rates and short irradiation times, but it gives less spectacular results for recording dose distributions at low ^60^Co irradiation dose rates and long irradiation times. The NBT-cotton dosimeter requires further optimization of its structure to approach the quality of 2D measurement, which is the case of EB irradiation.

## 4. Conclusions

This work reports on the use of a 2D radiochromic NBT-cotton dosimeter to measure the dose distribution of ionizing radiation for gamma radiation emitted by ^60^Co. The cotton fabric was volume modified by the padding-squeezing-drying method using an NBT solution. In consequence, yellow NBT-cotton dosimeter samples were obtained, which were ready for use immediately after production. The storage conditions of the dosimeter were determined in a preliminary study. The main conclusion is that the stability of NBT-cotton is related to both the access to daylight and the storage temperature. The most influential factor is exposure to daylight, while changes in storage temperature have a much smaller effect on the samples. The best option is to protect the dosimeter samples from daylight and store them in a refrigerator. Samples stored in this way can still be used 150 days after preparation. Irradiation of samples leads to a color change from yellowish to purple-brown, and samples are saturated after absorption of more than 32 kGy. The tests of NBT-cotton described in this work were related to the use of the properties of this dosimeter, which include shape adaptability and flexibility, enabling the measurement of 2D dose distribution on non-planar surfaces. The obtained results proved the potential of this dosimeter but also indicated a significant influence of the fabric structure on the quality of the results and the influence of air (oxygen) on color changes in the case of long-term irradiation at relatively low dose rates. This dosimeter is now ready for use in EB irradiations but requires further optimization of the structure in the case of ^60^Co irradiations. Due to the dose-response characteristics of NBT-cotton (the kGy dose response in particular), its current potential applications include industrial sterilization and may find application in nuclear power plant facilities. Optimization of the dosimeter structure, both the weave pattern, the stability of the radiation-sensitive compound, and the increase in dose sensitivity, may extend its application to 2D dose distribution measurements in radiotherapy, including in vivo measurements. Further studies after design optimization will include testing this flexible dosimeter for dose distribution measurements on complex, non-planar medical implant surfaces.

## Figures and Tables

**Table 1:** Percentage change in reflectance measurements (at 550 nm) for unirradiated NBT-cotton samples (1% NBT concentration used to produce NBT-cotton samples) stored at various temperatures and with or without daylight. Changes are reported relative to the sample just after preparation.

No.	Storage Conditions	Time [Days]				
		1	5	10	30	150
1	20–23 °C	13.10%	15.46%	24.30%	43.79%	54.19%
2	20–23 °C, covered with aluminum foil	1.27%	3.66%	4.17%	11.39%	23.93%
3	2–4 °C, covered with aluminum foil	0.62%	1.43%	1.56%	3.01%	6.72%
4	−17–−20 °C, covered with aluminum foil	0.88%	2.96%	3.66%	4.18%	4.25%

**Table 2:** Mean doses measured for ROIs indicated in Figure 8A by NBT-cotton dosimeter (2D measurements) placed on the surfaces of different metal alloys served for producing implants. In parentheses are doses measured by B3 WINdose film dosimeters (1D measurement).

Type of Alloy	8 mm Diameter Rod Made of a CoCrMo Alloy		16 mm Diameter Rod Made of a CoCrMo Alloy		16 mm Diameter Rod Made of Stainless Steel (316L)		Eleven 16 mm Diameter and 3 mm-High Cylinders Made of a TiVa and TiNb Alloy	
Dose (Irradiation Time: 17 h)	Front	Back	Front	Back	Front	Back	Front	Back
Minimum dose [kGy]	10.1	7.8	9.9	5.5	10.9	6.4	9.5	6.2
Maximum dose [kGy]	13.3	9.9	14.3	9.4	15.4	8.6	17.9	10.3
Mean dose [kGy]	11.8 (10.9)	8.3 (6.2)	12.3 (11.2)	6.8 (4.8)	13.0 (11.2)	7.1 (4.2)	11.9 (9.9)	7.3 (4.7)
Std [kGy]	0.8	0.4	0.8	0.5	1.1	0.4	1.0	0.5

**Table 3:** Comparison of doses measured with 2D NBT-cotton and 1D film dosimeters (B3 WINdose Dosimeters, GEX Corporation, USA). The doses for the NBT-cotton dosimeters are for ROIs indicated in Figure 9.

Y Distance [mm]	Dose Measured with NBT-Cotton [kGy]	Dose Measured with Film Dosimeters [kGy]
−20	13.7 ± 0.8	11.8
−39	11.0 ± 0.4	10.7
−55	11.5 ± 0.6	9.9
−73	10.7 ± 0.5	9.6
−92.5	10.0 ± 0.4	8.8
−110	9.8 ± 0.4	8.2
−130	8.4 ± 0.8	6.1

## Data Availability

The original contributions presented in the study are included in the article, further inquiries can be directed to the corresponding authors.
